# Aflatoxin Contamination: An Overview on Health Issues, Detection and Management Strategies

**DOI:** 10.3390/toxins15040246

**Published:** 2023-03-28

**Authors:** Meera Mohamed Alameri, Amanda Shen-Yee Kong, Mariam Nasser Aljaafari, Hajer Al Ali, Khadija Eid, Maryam Al Sallagi, Wan-Hee Cheng, Aisha Abushelaibi, Swee-Hua Erin Lim, Jiun-Yan Loh, Kok-Song Lai

**Affiliations:** 1 Health Sciences Division, Abu Dhabi Women’s College, Higher Colleges of Technology, Abu Dhabi 41012, United Arab Emirates; 2 School of Biosciences, University of Nottingham Malaysia, Jalan Broga, Semenyih 43500, Selangor, Malaysia; 3 Food Safety Department, Dubai Municipality, Dubai P.O. Box 67, United Arab Emirates; 4 National Accreditation Department, Ministry of Industry and Advanced Technology, Dubai P.O. Box 48666, United Arab Emirates; 5 Faculty Health and Life Sciences, INTI International University, Persiaran Perdana BBN, Putra Nilai, Nilai 71800, Negeri Sembilan, Malaysia; 6 Office of the Executive Campus Director, Abu Dhabi Colleges, Higher Colleges of Technology, Abu Dhabi 41012, United Arab Emirates; 7 Centre of Research for Advanced Aquaculture (CORAA), UCSI University, Kuala Lumpur 56000, Selangor, Malaysia

**Keywords:** *Aspergillus flavus*, *Aspergillus parasiticus*, aflatoxin exposure, liver cancer, oxidative stress, hormonal changes, food safety

## Abstract

Aflatoxins (AFs) represent one of the main mycotoxins produced by Aspergillus flavus and Aspergillus parasiticus, with the most prevalent and lethal subtypes being AFB1, AFB2, AFG1, and AFG2. AFs are responsible for causing significant public health issues and economic concerns that affect consumers and farmers globally. Chronic exposure to AFs has been linked to liver cancer, oxidative stress, and fetal growth abnormalities among other health-related risks. Although there are various technologies, such as physical, chemical, and biological controls that have been employed to alleviate the toxic effects of AF, there is still no clearly elucidated universal method available to reduce AF levels in food and feed; the only mitigation is early detection of the toxin in the management of AF contamination. Numerous detection methods, including cultures, molecular techniques, immunochemical, electrochemical immunosensor, chromatographic, and spectroscopic means, are used to determine AF contamination in agricultural products. Recent research has shown that incorporating crops with higher resistance, such as sorghum, into animal feed can reduce the risk of AF contamination in milk and cheese. This review provides a current overview of the health-related risks of chronic dietary AF exposure, recent detection techniques, and management strategies to guide future researchers in developing better detection and management strategies for this toxin.

## 1. Introduction

Mycotoxins present a detrimental threat to human and livestock health by contaminating various food substances and agricultural products [1]. The Food and Agricultural Organization of the United Nations has reported that mycotoxins affected at least 25% of the global food crop in 2004 [2]. Consumption of mycotoxin-contaminated products can lead to acute and chronic toxicity [3]. Aflatoxins (AFs), produced primarily by Aspergillus flavus and Aspergillus parasiticus, represent one of the most poisonous mycotoxins [4].

AFs can be classified into six types: AFs B1 (AFB1), B2 (AFB2), G1 (AFG1), G2 (AFG2), M1 (AFM1), and M2 (AFM2) [5]. Of these, AFB1 and AFB2 are produced by A. flavus, whereas AFG1 and AFG2 are released by A. parasiticus [6]. In contrast, AFM1 has been considered the hydroxylation byproduct of AFB1 in the liver of lactating dairy cows after the ingestion of contaminated feed [7]. AFB1, AFB2, AFG1, and AFG2 are primarily found in food crops, whereas AFM1 (a metabolite of B1) and AFM2 are commonly found in animal by-products, such as milk and dairy products [8,9,10].

Food products, such as grains, tree nuts, oilseeds, and spices, are frequently affected by AFs under warm and humid storage conditions [4]. Other environmental factors (temperature, relative humidity, rainfall, soil type, and evapotranspiration) and pre- and post-harvest management practices (cropping, timely harvesting, drying, sorting, storage conditions, and transportation) may also contribute to fungal proliferation and trigger subsequent mycotoxin excretion [11]. Mainly, AF contamination affects agricultural products in African and Southeast Asia countries due to their climatic conditions [12], where hot and humid tropical and subtropical climates with mean annual rainfalls > 700 mm provide ideal conditions conducive to the growth of molds and post-harvest products stored under conditions with high relative humidity and poor aeration that promote fungal growth [13,14,15]. However, with increasing global warming, AF is now becoming a threat in previously unaffected countries, including Europe [16].

Despite numerous works of literature on AFs contamination in the current field, there is still limited documented evidence on the level of AF awareness and the food handling practices that can help minimize AF food and feed contamination. There is an urgent need to carry out interventions to protect food safety and security. Thus, in this review, we focus on the health-related risks of human and livestock dietary exposure to AFs, along with the various methods used to detect, control, and manage AFs.

## 2. Impact on Human and Animal Health

AF can have a negative impact on the physiological status of humans and animals by causing DNA damage, cancer, and developmental abnormalities in embryos under long-term exposure [17]. Upon consumption, the alternating groups of carbonyl and methylene, called polyketides, are absorbed, modified, and transferred to different parts of the body [6]. Long-term exposure to AFs can result in aflatoxicosis, an acute poisoning that can be life-threatening and predominantly causes liver damage [4]. Research has shown that children receive the most significant exposure to all AF-contaminated food types, followed by adolescents and adults, who were the least-exposed group [18]. The existing evidence suggests that infants and young children have a greater possibility of experiencing the deleterious effects of mycotoxins due to their immature metabolic pathways, higher intake-to-body weight ratio, greater metabolic rates, and lesser detoxification capability relative to adults [19,20]. Based on the reported epidemiological studies, AFB1 is the most dangerous AF [21]. Ezekiel and colleagues (2021) determined the liver cancer risk for households that consume cereals and nuts regularly. They observed that the liver cancer risk from AFB1 exposure in children was twice that of adolescents and six times that of adults. In contrast, Nabizadeh and colleagues (2018) revealed no considerable differences in the margin of exposure between adults and children in the Zanjan Province, Iran, despite both groups being at significant risk of liver cancer due to AFB2 from the consumption of unrefined olive oil [22]. It is suggested that the reason may be linked to a lower daily intake of olive oil in children.

Similarly, Milićević and colleagues (2021) found that pasteurized and UHT milk had the highest level of contamination (79%) and the greatest mean concentration of AFM1 (22.34 ± 0.02 ng kg^−1^), while cheese had the lowest mean concentration (1.36 ± 0.01 ng kg^−1^) [20]. The main contributor to the risk of hepatocellular carcinoma (HCC) resulting from AFM1 exposure was the consumption of milk products, in the form of pasteurized and UHT milk, with estimated cases of 0.00038 and 0.00039 per 100,000 individuals per year for the lower bound and upper bound scenarios, respectively. Interestingly, the age group of 1–3 years was associated with the highest risk of HCC (0.00034), indicating no health risk for the groups assessed. Toddlers were estimated to have a higher daily exposure to AFM1 in milk compared to children aged 3–9 years, with an estimated daily intake of 0.164 and 0.193 ng kg^−1^ bw day^−1^ for the lower and upper bound exposure scenarios, respectively.

In a recent study by Arak and colleagues (2021), the consumption of raw rice grain powder and methanol extract containing AFs caused a significant increase in lactate dehydrogenase activity in experimental ducklings [23]. Histopathological examinations revealed an accumulation of large fat droplets and hepatocyte cell swelling in the ducklings exposed to dietary AFs. The presence of AFB1, in combination with biomolecules, led to liver damage and impaired liver metabolic functions. Similarly, de Freitas Souza and colleagues (2019) observed hepatocyte anisocytosis, moderate fat infiltration, apoptosis, and the multifocal necrosis of hepatocytes in silver catfish at 5 days post-feeding with an AFB diet [24]. The severity of toxic hepatitis significantly increased by day 10 post-feeding.

Rotimi and colleagues (2021) investigated the effects of prenatal exposure to AFB1 in mice and its impact on weight, lipid levels, and hormone levels in the offspring [25]. The results indicated that both low and high levels of prenatal AFB1 exposure caused a significant reduction in weight and a decrease in cholesterol levels, accompanied by an increase in triglyceride levels. Furthermore, the weight of the newborn mice was reduced, and weight gain was affected even after the exposure was withdrawn. Additionally, hormonal changes were observed in male and female mice, including decreased levels of testosterone, progesterone, and luteinizing hormone.

Morales-Moo and colleagues (2020) revealed that AFB1 was detected in at least 47% of popcorn samples, highlighting the potential risk associated with consuming this popular snack [26]. The risk of liver cancer due to the consumption of AF-contaminated popcorn was found to be 0.993 cancers/year per 100,000 females, while for males, the average risk was 0.137 cancers/year per 100,000. Notably, males under the age of 18 carried the highest risk at 0.137 cases per 100,000 persons. The reasons for the presence of AF in processed foods remain poorly understood; however, it is thought to be linked to the limitations of the current cooking processes that can only partially destroy AF, resulting in residual contamination [27].

Recently, Hatipoglu and colleagues (2022) demonstrated that AFB1 can induce oxidative stress by generating reactive oxygen species (ROS) and causing lipid peroxidation [28]. This leads to a significantly increased level of malondialdehyde (MDA) and decreased activities of glutathione (GSH) and superoxide dismutase (SOD). Additionally, AFB1 triggers the release of pro-inflammatory cytokines, including tumor necrosis factor-a (TNF-a), interleukin-1b (IL-1b), and interleukin-6 (IL-6). Abnormal liver function tests that included high levels of AST and ALT further explained the loss of hepatocyte structural integrity. AFB1 can disrupt cell membrane permeability and the mitochondrial membrane in hepatocytes, leading to liver damage.

AF derivatives, such as AFM1, can contaminate milk products through animal excretion [4]. Islam and colleagues (2021) reported that over half of the 62 human breast milk samples from the Bangladesh cohort were contaminated with AFM1 due to maternal consumption of AFB1-contaminated food [29]. Using enzyme-linked immunosorbent assay (ELISA), the presence of AFM1 was detected in 51.6% of the samples, with an average daily intake of AFM1 in human newborns of 0.49 ng/kg b.w./day. This investigation was the first of its kind to determine the occurrence of AFM1 in human breast milk in Bangladesh, where limited data exist on AF occurrence in food commodities. Further studies involving a larger cohort are proposed to gain more insight into the extent of infant exposure through maternal milk in Bangladesh.

Interestingly, Njombwa and colleagues (2021) observed a very low incidence of AFM1-induced HCC in children, at 0.038 cases/100,000 individuals, and adults, at 0.023 cases/100,000 individuals, despite their high consumption of raw milk, at 300 mL/day and 541 mL/day, respectively [30]. According to the eighty-third report of the World Health Organization and the Food and Agriculture Organization of the United Nations, AFM1 only contributed a small amount (<1%) to AF-induced cancer risk for the general population compared to AFB and AFG [31]. Additionally, Conteçotto and colleagues (2021) revealed the risk of HCC in children was at 0.0015 to 0.0045 cases/100.000 individuals from the consumption of ultra-high temperature milk, powdered milk, and infant formula [32]. The average number of HCC cases associated with AFM1 exposure was reported between 0.0027 and 0.0029 cases/100,000 individuals.

**Figure 1:**
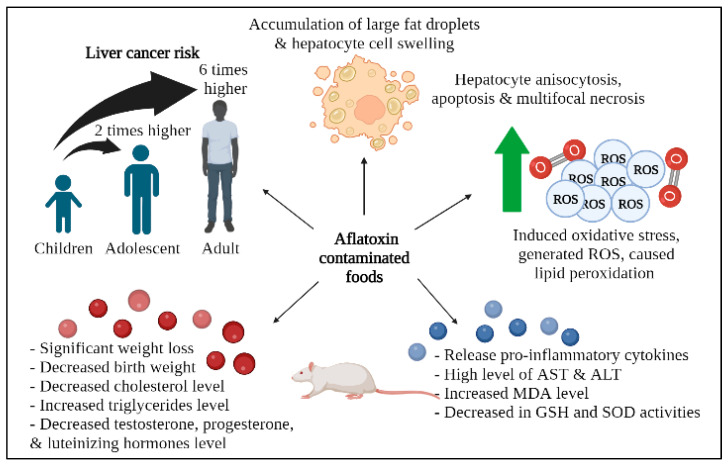
A schematic representation of health-related risks for humans and animals upon dietary AF exposure. Generally, exposure to AF can increase liver cancer risk, induce oxidative stress, manipulate the biochemical profile, and cause changes to hepatocyte cells. Created with BioRender.com (accessed on 7 November 2022).

Currently, there is a lack of literature available on the levels of AFM2 in naturally contaminated cheese and the associated health risks of its consumption. Despite this, some studies have investigated the distribution of AFM2 in mozzarella cheese made with buffalo milk. Pietri and colleagues (2003) and Fedele and colleagues (2007) both reported lower levels of AFM2 compared to AFM1 and suggested that this may be due to a lower interaction of AFM2 with casein [33,34]. Figure 1 shows the schematic representation of health-related risks for humans and animals upon dietary AF exposure, while Table 1 summarizes the impacts of AF on human and animal health, as appraised in this review.

## 3. Detection of Aflatoxin

Apart from their impact on human health, AFs also lead to significant economic losses due to the widespread contamination of food products. Therefore, the detection and quantification of AFs in food and feed are crucial for ensuring safety. Several methods are usually employed for the quantification of AFs in food commodities.

### 3.1. Culture-Based Techniques

Different culture media, such as coconut agar medium (CAM), coconut milk agar (CMA), yeast extract sucrose (YES) medium, and AF-producing ability (APA) media, can be used to distinguish between toxigenic and atoxigenic strains of *A. flavus* based on their morphological characteristics [35,36,37]. To differentiate between these strains, UV light of 365 nm wavelength was used to observe a fluorescent ring surrounding the *A. flavus* colony on CAM, CMA, and YES media amended with 3% methyl-cyclodextrin, while the atoxigenic isolates showed no fluorescence. Research conducted by Wang and colleagues (2019) investigated the AF production of *A. flavus* at different temperatures and media. They observed that AF production was highest in solid media at 28 °C and 37 °C and in liquid media at 28 °C [38]. The reason for more AF production in solid media can be attributed to the association of multiple metabolic pathways.

### 3.2. Molecular-Based Techniques

Molecular-based techniques outperform culture-based techniques with their higher sensitivity, reproducibility, and reliability [39]. To amplify the genes involved in the AF biosynthesis pathway, various markers have been utilized. Multiplex and real-time PCR assays have been developed for this purpose, and they target genes, including *aflD*(*nor*), *omtA*, *aflM(ver)*, *aflR*, and *aflJ* [40]. Hu and colleagues (2021) developed a novel luminescence detection method that employs ATP-releasing nucleotides (ARNs) and AFB1 aptamer for AFB1 detection [41]. The authors synthesized two ARNs (dTP_4_A and dGP_4_A) without using ATP as the starting material, and the method provided a lower detection limit of 0.3 pM. Zhao and colleagues (2021) used a self-replicating catalyzed hairpin assembly method based on the formation of a helix DNA H1–H2 complex for AFB1 identification [42]. Within 15 min, AFB1 was detected with a detection limit of 0.13 ng/mL, as the DNA complex splits the double-stranded probe DNA, leading to the development of DNA replicas and fluorescence signals.

### 3.3. Immunochemical Methods

Immunochemical methods, such as ELISA, radioimmunoassay (RIA), and immunodipsticks utilize the specific binding of antigens and antibodies. In ELISA, enzymes are used to label antigens or antibodies that can be analyzed with specific substrates to improve their sensitivity, making it an easy and quick method for detecting AFs in crops and food products [43]. Three-dimensional structured AF can be differentiated by specific antibodies [44]. ELISA kits, such as Veratox^®^, are commonly used for AF measurement in different samples and can detect AF concentrations ranging from 5 to 50 ppb [45]. Azri and colleagues (2018) developed an ultrasensitive electrochemical immunosensor that utilized an indirect competitive ELISA to detect AFB1 [46]. The immunosensor had a detection limit of 0.3 pg/mL, with 4.78% reproducibility and 2.71% repeatability, using modified multi-walled carbon nanotubes/chitosan/screen-printed carbon electrodes.

Similarly, a competitive magnetic immunodetection assay was implemented by Pietschmann and colleagues (2020) for the detection and quantification of AFB1, but it has a detection limit of 1.1 ng/mL [47]. Recently, Peltomaa and colleagues (2022) developed a single-step immunoassay based on a monoclonal capture antibody and a recombinant anti-immunocomplex antibody fragment isolated from a synthetic antibody repertoire to detect AF in contaminated food samples [48]. This assay has a detection limit of 70 pg/mL and produces results within 15 min, utilizing a single incubation step where all three antibody reagents (biotinylated monoclonal antibody, anti-IC binder, and europium-labeled secondary antibody) are added simultaneously. The simple and rapid protocol of the established method makes it highly suitable for rapid testing or the high throughput screening of various food products for AF and other small molecule contaminants.

### 3.4. Electrochemical Immunosensors

Electrochemical immunosensors have gained recognition as a simple, inexpensive, and time-saving technique for detecting AFs [49,50]. These biosensor devices incorporate antibodies on a biorecognition layer and amplify signals using biosensor amplifiers to recognize and quantify the signals generated [51]. The electrochemical immunosensors function by restricting the antibodies on the electrode surface, and some use enzymes as biological agents to produce signals. Non-enzymatic electrochemical immunosensors are also available for analyzing AFs.

Abera and colleagues (2019) employed an electrochemical immunosensor technique to detect AFM1 in milk samples using biosensors made from versatile printing electrodes, such as insulators, conductors, and semiconductors, that work in tandem with single-walled carbon nanotubes and specific antibodies for higher sensitivity [50]. This technique could detect AFM1 at concentrations ranging from 0.01 to 1 g/L. Sojinrin and colleagues (2019) developed a rapid and sensitive gold nanoparticle (AuNP) immunochromatographic strip to detect AFB1 in peanuts, corn, rice, and bread samples [52]. The researchers used novel AuNPs-conjugated AFB1 antibody derivatives to develop a colorimetric assay in 96-well plates and lateral flow immunochromatographic assays (LFIAs) strips. The detection of AFB1 could be monitored via a visible color change from red to purple or blue, with a detection limit of 2 ng/mL in the 96-well plate assay and 10 ng/g in the LFIA. The researchers suggested smartphone-based LFIAs for AFB1 detection in food samples with a detection limit of 0.3 ng/g based on the results obtained.

### 3.5. Chromatographic Methods

Chromatographic techniques, such as HPLC, thin-layer chromatography (TLC), and LC-MS, are based on the physical interaction between a mobile phase (liquid or gaseous components) and a stationary phase (liquid or solid) [53]. Chromatography involves the separation of molecules in a mixture that is applied on a surface or into a solid, with the aid of a mobile phase. Due to differences in molecular weight, certain parts of the mixture remain in the stationary phase and move slowly through the chromatography system, while others rapidly pass into the mobile phase and leave the system more rapidly. To investigate the prevalence of toxigenic Aspergillus species in processed meat samples, Algammal and colleagues (2021) employed HPLC to sequence the aflR1 gene [54]. By combining phenotypic and molecular identification methods, the researchers successfully amplified the internal transcribed spacer region of A. flavus and detected AFB1 in 15% of the basturma samples. However, the application of the chromatographic methods was limited by their cumbersome procedures, the use of heavy equipment, and complex operations.

### 3.6. Spectroscopic Methods

Fluorescence spectrophotometry is a useful tool for detecting AF, as different fluorescent compounds emit energy at specific wavelengths. In less than 5 min, AFs can be quantified using fluorescence spectrophotometry within the range of 5 ppb to 5000 ppb [55]. Meanwhile, hyperspectral imaging (HSI) has emerged as a powerful technique that combines imaging, spectroscopy, and computer vision to provide both spatial and spectral information from a sample [56]. To rapidly detect AF, Zhong Zhi and colleagues (2020) employed a hyperspectral imaging method that exploited the ultraviolet fluorescence and superficial distribution of AF [57]. They proposed a machine learning detection method based on a support vector machine that combined the band index and narrow band. By comparing the AF concentration, they presented three fluorescence indexes based on the average gray value of the radiation index. The optimal wavelength, selected using Fisher’s discriminant, was 410–430 nm, which outperformed the other three band selection methods.

## 4. Management and Control Strategies

AF contamination in crops is a major concern, as it poses significant risks to production, food safety, public health, and the economy. To address this issue, various methods have been developed to reduce AF contamination in crops, including physical, chemical, and biological approaches [9]. Moreover, many countries have established stringent regulations for AFs in human food and animal feed to safeguard public health [58]. The acceptable limit of AF for human consumption ranges from 4 to 30 μg/kg [59]. The European Union has set the strictest safety levels, with AFB1 and total AFs not exceeding 2 g/kg and 4 g/kg, respectively, in any direct consumption product [60,61].

### 4.1. Physical Methods

Prevention measures, including the implementation of good agricultural and manufacturing practices and proper storage conditions, have been adopted to decrease AF contamination; however, these approaches are not always effective [17]. Physical methods, such as steam under pressure, dry roasting, and other cooking methods, have been found to be effective in controlling or reducing AF contamination in many crops [62]. High hydrostatic pressure and pulsed electric field treatments have been shown to significantly reduce AF levels in grape juice, with a shorter processing time compared to thermal processing, while maintaining nutritional quality and being more ecologically friendly [63]. When peanuts were exposed to 2.3 mW/cm^2^ UV-C irradiation coupled with an 11 rpm rotation for 2 h, the percentage of AFB1 degradation increased from 60.8 to 75.0 pmol/g/h [64]. Similarly, gamma irradiation, at a dose of 6 kGy, effectively reduced the AFB1 level [65]. Zhang and colleagues (2018) suggested that gamma irradiation at a dose above 10 kGy may significantly decrease the AFB1 concentration in soybeans [66].

### 4.2. Chemical Methods

When used appropriately, certain chemicals and gases have been shown to reduce the growth and production of AFs. These include acids, alkalis, oxidizing agents, aldehydes, and some gasses [67]. Chemicals, such as sodium bisulfite, calcium hydroxide, formaldehyde, sodium hypochlorite, sodium borate, and sorbents, have been found to significantly reduce AF levels in various food products [68]. Jubeen and colleagues (2020) reported the production of AFD1, a less toxic product, when citric and lactic acids were used to convert AFB1 via hydrolysis of the lactone ring [69]. Citric acids were found to have better AF detoxification results compared to conventional methods. A maximum reduction of 99% of AFB1 was observed in walnuts treated with 9% aqueous citric acid for 15 min. Similarly, Dhanshetty and colleagues (2021) demonstrated that roasting, in the presence of sodium chloride and citric acid, reduced AFB1 contents the most [70]. Cooking under pressure, with the presence of sodium chloride and citric acid, also significantly reduced AF levels compared to the frying method.

Ozone gas has shown promise for reducing AFB1 levels in poultry feed [71]. Torlak and colleagues (2016) reported a significant reduction in AFB1 levels of 86.4% following ozone treatment for 240 min. However, the high cost of ozonation may limit its application in post-harvest processes.

### 4.3. Biological Factors

Biological control technologies have employed selected microorganisms, such as bacteria, yeasts, and nontoxigenic molds, to reduce AF contamination in pre- and post-harvest agricultural production [17]. The adverse effects of these microorganisms, such as space and nutrient competition, or biological interactions, such as antibiosis, are used by researchers to control AF. In countries producing maize, non-toxigenic strains of *A. flavus* (Mytoolbox Af01) have been employed as a biological control to reduce AF levels, relying on the competitive role between atoxigenic and toxigenic strains [72]. This method successfully reduced the level of AFs in maize kernels by 51–83%. Ali and colleagues (2021) used various bacteria, including *Enterococcus* sp., *Bacillus* sp., *Stenotrophomonas* sp., and *Pseudomonas* sp., to reduce AF levels [73]. Their research revealed that Pseudomonas fluorescens MN256402.1 can reduce AFB1, AFB2, and AFG2 by 99% and AFG1 by 100%. Moreover, this is the first report of *Enterococcus casseliflavus* SA21, B. haynseii SA22, B. tequilensis S18, and B. amyloliquefaciens S8C exhibiting AF degradation functions.

One potential strategy to prevent the production of AF in animal feed is to incorporate crops with higher resistance, such as sorghum, into the feed [74]. This approach involves natural methods to control the growth and spread of *Aspergillus* fungi that produce AFs. Buonaiuto and colleagues (2021) investigated the effects of replacing corn with finely ground sorghum meal in dairy cow diets in the Parmigiano Reggiano region and reported no adverse effects on herd productivity, milk quality, or cheese yield [75]. Sorghum meal is a viable substitute for corn that can contribute to the economic sustainability of farms by increasing crop production. Compared to other cereals, sorghum exhibits resistance to environmental stressors, including dry, saline, and hot conditions. By incorporating sorghum grain into animal feed, the risk of AF contamination in milk and cheese can be reduced, resulting in safer food products for consumers.

## 5. Future Prospect

The future of food safety in relation to AF mainly depends on sharpening our focus on the farmer group. Farmers are the first-hand group that will be dealing with AF and should be responsible for permitting a more thorough examination of farming practices. However, research has shown that more than 70% of farmers had zero knowledge about AFs [76]. Factors, such as education level, specialization, and how many years of experience the farmers had in raising livestock, bore a significant impact on the farmers’ awareness of AF. Authorities should, therefore, find ways to raise AF awareness in this group. Some examples are making exposure to life sciences mandatory for all farmers and enforcing AF knowledge in the school curriculum. Farmers should know the details of how, when, and where they should use biocontrol and other relevant methods to prevent the spread of toxins.

Increased customer demand and investments in technology will likely incentivize processors to produce alternative products from contaminated food and assign economic value to AF-contaminated food products [77]. Nevertheless, there is still a need for novel technologies that can help elucidate the possible effects of climate change on AF contamination, including the collection of data and the monitoring of AFs and/or AF-producing fungi.

## 6. Conclusions

Our review paper recognizes the potential limitations with regard to the availability of the included studies and acknowledges the possibility that some relevant studies and important findings may have been overlooked. In addition, this review is limited to the latest articles published between 2018 and 2023. AFs are toxic secondary metabolites synthesized by *Aspergillus* species, particularly *A. flavus* and *A. parasiticus.* Technological advancements have facilitated the study of AF structures and biosynthetic pathways, enabling early detection. Various management techniques have been utilized worldwide to control AFs. To safeguard consumer health, biocontrol methods should be implemented in tandem with other physical and chemical approaches, as well as improvements in storage and packaging materials. This review presents an updated literature study on AFs, which can be used to aid future researchers in developing mitigating strategies to better detect and manage this toxin.

## Figures and Tables

**Table 1:** A summary of AF impacts on human and animal health.

Type of Aflatoxins	Country	Host/Food Commodities	Detection Method	Study Subjects	Major Findings	References
AFB1	North Central Nigeria	Cereals, millet, rice, sorghum, nuts, and legumes	Liquid chromatography tandem mass spectrometry (LC-MS)	Average household population, which regularly consumes cereals and nuts	Liver cancer risk for AFB1 exposure in children was twice that of adolescents and four times that of adults.	[18]
AFB2	Iran	Sunflower oil, canola oil, refined olive oil, unrefined olive oil, frying oil, and blend oil	High-performance liquid chromatography (HPLC) with a fluorescence detector	Children and adult populations exposed to contaminated edible oils	There was no considerable difference in the margin of exposure between adults and children in the Zanjan Province, Iran despite both groups being at significant risk of liver cancer due to AFB2 from the consumption of unrefined olive oil. It is suggested that the reason may be linked to a lower daily intake of olive oil in children.	[22]
AFM1	Serbia	Milk, dairy products, and infant formula	ELISA and LC-MS/MS analysis	Toddlers (1–3 years) and children (3–9 years)	Pasteurized and UHT milk had the highest level of contamination (79%) and the greatest mean concentration of AFM1 (22.34 ± 0.02 ng kg^−1^), while cheese had the lowest mean concentration (1.36 ± 0.01 ng kg^−1^). The main contributor to the risk of HCC resulting from AFM1 exposure was the consumption of milk products, in the form of pasteurized and UHT milk, with estimated cases of 0.00038 and 0.00039 per 100,000 individuals per year for the lower bound and upper bound scenarios, respectively. The age group of 1–3 years was associated with the highest risk of HCC (0.00034), indicating no health risks for the groups assessed. Toddlers were estimated to have a higher daily intake of AFM1 in milk as compared to children aged 3–9 years, with an estimated daily intake of 0.164 and 0.193 ng kg^−1^ bw day^−1^ for the lower and upper bound exposure scenarios, respectively.	[20]
AFB1	Iran	Rice grains (contaminated rice grain powder, methanol extract of contaminated rice grains)	HPLC	Four-day-old Pekin ducklings	The consumption of raw rice grain powder and methanol extract containing AFs caused a significant increase in lactate dehydrogenase activity in experimental ducklings. Histopathological examinations revealed an accumulation of large fat droplets and hepatocyte cell swelling in the ducklings exposed to dietary AFs. The presence of AFB1, in combination with biomolecules, led to liver damage and impaired liver metabolic functions.	[23]
AFB	Brazil	Rice	LC-MS, equipped with a turbo ion spray electron spray ionization source at atmospheric pressure, and an Agilent chromatography system	Silver catfish (*Rhamdia quelen*)	Hepatocyte anisocytosis, moderate fat infiltration, apoptosis, and the multifocal necrosis of hepatocytes were observed in silver catfish at 5 days post-feeding with an AFB diet. The severity of toxic hepatitis significantly increased by day 10 post-feeding.	[24]
AFB1	Nigeria	Not stated	Not stated	Male and female Wistar rats	Both low and high levels of prenatal AFB1 exposure caused a significant reduction in weight and a decrease in cholesterol levels, accompanied by an increase in triglyceride levels. The weight of newborn mice was reduced, and weight gain was affected even after the withdrawal of exposure. Hormonal changes were observed in male and female mice, including decreased levels of testosterone, progesterone, and luteinizing hormone.	[25]
AFB1	Mexico	Popcorn (*Zea mays everta*)	HPLC equipped with an isocratic pump, a fluorescence detector, and an autosampler	Women and men in the city of Veracruz (food frequency questionnaires)	AFB1 was detected in at least 47% of the popcorn samples. The risk of liver cancer due to the consumption of AF-contaminated popcorn was found to be 0.993 cancers/year per 100,000 females, while for males, the average risk was 0.137 cancers/year per 100,000. Notably, males under the age of 18 carried the highest risk, at 0.137 cases per 100,000 persons.	[26]
AFB1	Turkey	Not stated	Not stated	Wister albino rats	AFB1 induced oxidative stress by generating ROS and causing lipid peroxidation. This leads to a significantly increased level of MDA and decreased activities of GSH and SOD. Additionally, AFB1 triggers the release of pro-inflammatory cytokines, including TNF-a, IL-1b, and IL-6. Abnormal liver function tests that included high levels of AST and ALT further explained the loss of hepatocyte structural integrity. AFB1 can disrupt the cell membrane permeability and the mitochondrial membrane in hepatocytes, leading to liver damage.	[28]
AFM1	Bangladesh	Human breast milk samples	Competitive ELISA	Nursing mothers and nursing babies	Maternal consumption of AFB1-contaminated food led to more than half of the 62 human breast milk samples from the Bangladesh cohort that was contaminated with AFM1. Using ELISA, the presence of AFM1 was detected in 51.6% of the samples, with an average daily intake of AFM1 in human newborns of 0.49 ng/kg b.w./day.	[29]
AFM1	Malawi	Raw milk samples	VICAM aflatest fluorometry	Adults and children from small-scale dairy farming households	A very low incidence of AFM1-induced HCC was observed in children, at 0.038 cases/100,000 individuals, and adults, at 0.023 cases/100,000 individuals, despite their high consumption of raw milk, at 300 mL/day and 541 mL/day, respectively.	[30]
AFM1	Brazil	Ultra-high temperature milk, powdered milk, and infant formula	HPLC with a fluorescence detector	Children from a child education center (food frequency questionnaires)	The risk of HCC in children was identified at 0.0015 to 0.0045 cases/100.000 individuals from the consumption of ultra-high temperature milk, powdered milk, and infant formula. The average number of HCC cases associated with AFM1 exposure was reported to be between 0.0027 and 0.0029 cases/100,000 individuals.	[32]

## Data Availability

Not applicable.
